# Mind the gender gap: A Scoping Review of Inequalities in Dental Science and Leadership Short title: Mind the gender gap

**DOI:** 10.1590/0103-644020256716

**Published:** 2026-04-10

**Authors:** Luisa Gatti-Reis, Maisa Costa Tavares, Lucas Guimarães Abreu, Isabela Almeida Pordeus, Saul Martins Paiva

**Affiliations:** 1 Department of Paediatric Dentistry, Universidade Federal de Minas Gerais (UFMG), Belo Horizonte, MG, Brazil.

**Keywords:** dentistry, dental research, gender equity, sexism, intersectional framework

## Abstract

This study aimed to carry out a scoping review to map the scope and nature of the literature on gender equity and discrimination in dental research and leadership, using an intersectional approach. The protocol of this scoping review is available online in the Open Science Framework platform. The literature search was carried out in February 2024. The terms of the search strategy encompassed three concept groups: gender inequality, the outcomes assessed, and dental science. PubMed, Scopus, Web of Science, and EMBASE were consulted. Two independent reviewers assessed the retrieved records. The initial search returned 2,322 references; after screening, 362 duplicates were removed, and 1,923 records were excluded because titles and abstracts did not meet the eligibility criteria. Inter-examiner agreement between reviewers was high (kappa = 0.931). For the updated search, 424 references were retrieved, and 52 duplicates were removed. After screening the titles and abstracts of the remaining 372 references, 11 were selected for full-text screening, of which 10 were included in the qualitative synthesis. Combining the two searches, 35 references were included in this scoping review. The studies included in this review were conducted by authors from multiple countries: Brazil, the United States, Canada, Saudi Arabia, Iran, Australia, and Austria, published between 1998 and 2024. The included studies showed persistent gender inequalities in dental research across different levels, particularly in leadership positions. There is a need to encourage further research on this topic under an intersectionality lens, on a broader scope of concepts, and to test interventions.

## Resumo

Este estudo tem como tema a desigualdade de gênero na pesquisa odontológica. O objetivo foi realizar uma revisão de escopo para mapear o escopo e a natureza da literatura existente sobre equidade de gênero e discriminação na pesquisa e liderança odontológica, usando uma abordagem interseccional. O protocolo desta revisão de escopo está disponível online na plataforma *Open Science Framework*. A busca bibliográfica foi realizada em fevereiro de 2024. Os termos da estratégia de busca abrangeram três grupos conceituais: a desigualdade de gênero, os desfechos avaliados e a ciência odontológica. PubMed, Scopus, Web of Science e EMBASE foram consultados. Dois revisores independentes avaliaram os registros recuperados. A busca inicial retornou 2.322 referências; após a triagem, 362 duplicatas foram removidas e 1.923 registros foram excluídos porque os títulos e resumos não atendiam aos critérios de elegibilidade. A concordância interexaminadores entre os revisores foi alta (kappa = 0,931). Para a busca atualizada, 424 referências foram recuperadas e 52 duplicatas foram removidas. Após a triagem dos títulos e resumos das 372 referências restantes, 11 foram selecionadas para triagem de texto completo, das quais 10 foram incluídas na síntese qualitativa. Combinando as duas buscas, 35 referências foram incluídas nesta revisão de escopo. Os estudos incluídos nesta revisão foram conduzidos por autores de vários países; Brasil, Estados Unidos, Canadá, Arábia Saudita, Irã, Austrália e Áustria, publicados entre 1998 e 2024. Os estudos incluídos mostraram que existem desigualdades de gênero persistentes na pesquisa odontológica em diferentes níveis, particularmente em posições de liderança. Há uma necessidade de incentivar mais pesquisas sobre este tópico sob uma perpectiva interseccional, em um escopo mais amplo de conceitos e testando intervenções.

**Figure:**
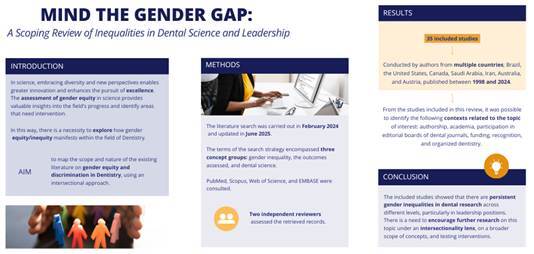

## Introduction

Historically, women have been primarily responsible for domestic tasks and family care, a role often not shared equally with men^1^ ^,^ ^2^. Currently, women are estimated to be responsible for approximately three-quarters of unpaid labor worldwide^1^. This socially prescribed gender role negatively influences the time and resources women can dedicate to their professional work, often hindering their productivity and career advancement, particularly for female scientists^3^.

Indeed, women remain underrepresented in several fields, particularly in STEM (Science, Technology, Engineering, and Mathematics) disciplines^4^. In the dental field, female participation has been increasing worldwide^5^; however, women continue to face significant barriers to career progression^6^. The gender gap in research may stem from different factors, including lower work performance, individual bias, and systemic bias^7^. A plausible explanation for this lower performance is associated with the traditional social expectations that assign women the primary responsibility for caregiving and household management, roles they tend to fulfill more frequently than their male counterparts^6^. Individual bias refers to individual assessments that occur in a conscious or unconscious manner that may affect one's evaluations and judgment^7^. For instance, in Dental research, both men and women assessed the curriculum vitae (CV) of candidates in a simulated post-doctoral selection process and assigned higher scores to CVs of male candidates in comparison to CVs of female candidates, even though the CVs were identical^6^. Systemic bias indicates the structural organization of selection processes that may inadvertently favor male researchers due to advantage accumulation, such as giving higher scores to those who have already had a higher number of publications or who have received more grant funding^7^. 

The challenges faced by female scientists in the Dental field range from biased evaluation in selection processes to limited representation in leadership roles^6^ ^,^ ^7^ ^,^ ^8^ ^,^ ^9^. There are persistent gender inequalities in editorial boards of prominent scientific journals^8^, in deanship of academic institutions^8^, and in the speakers invited to speak at major conferences^10^, with underrepresentation of female scientists. Additionally, gender inequality is evident in the authorship of high-impact publications within the field of Dentistry^11^.

In science, embracing diversity and new perspectives enables greater innovation and enhances the pursuit of excellence^5^ ^,^ ^12^. The assessment of gender equity in science provides valuable insights into the field's progress and identifies areas that need intervention^2^.

In this way, there is a necessity to explore how gender equity/inequity manifests within the field of Dentistry. A scoping review serves as an effective tool for synthesizing evidence, answering exploratory research questions, mapping existing concepts, gaps, and future research directions^13^. This review aims to map the scope and nature of the existing literature on gender equity and discrimination in Dentistry, using an intersectional approach.

## Material and methods

The reporting of this review was carried out according to the guidelines provided in the extension of the Preferred Reporting Items for Systematic Reviews and Meta-Analyses (PRISMA) for scoping reviews: PRISMA-ScR^14^. The protocol of this scoping review is available online in the Open Science Framework platform (https://osf.io/56pdf/?view_only=c623ba9cb10042b4b8722c5609b0c053).

### Eligibility criteria

The research question was formulated based on the objective. The “PCC” mnemonic was used, using the criteria ‘Participants, Concept and Context’ - PCC, as displayed in Table 1 ^15^. The research questions of this scoping review were: primary question 1) “What findings does the literature present regarding gender equity/inequity in dental science leadership? Moreover, a secondary question 2) “How does gender intersect with other relevant social identities to influence the observed outcomes?"

For this review, the following study designs were included: a) observational studies (cross-sectional, case-control, and longitudinal) and b) bibliometric analysis. There were no restrictions regarding language or year of publication. Literature review studies were ineligible.

**Table 1:** Definition of the criteria: 'Participants, Concept and Context'.

Acronym	Meaning	Study definition
P	Participants	Dental researchers
C	Concept	Gender inequality, gender discrimination
C	Context	Leadership in dental science, including: research, publication, presentation, award, prize, intellectual property ownership, funding, grant, scholarship, leadership, editor, speaker, presenter, authorship, collaboration, mentorship

### Information sources

The PubMed, Scopus, Web of Science, and EMBASE databases were consulted for this review. We did not search the grey literature, as the review aimed to map the existing published literature on gender inequality in dental science.

### Search

The literature search was carried out in February 2024. An update was performed in June 2025. The search strategy was formulated based on the research question, using terms identified as Medical Subject Headings and text words linked with the Boolean operators “OR” and “AND”. The terms encompassed three concept groups: gender inequality, the outcomes assessed, and dental science. The search strategy used in PubMed was as follows: Gender* OR equit* OR inequit* OR equal* OR inequalit* OR “implicit bias” OR “unconscious bias” OR “gender bias” OR “gender gap” OR disparit* OR parity OR disparit* OR diversit* OR underrepresent* OR under-represent* AND productivity OR award* OR prize OR citation* OR dean* OR publication* OR presentation* OR speaker* OR “intellectual property ownership” OR researcher OR leader* OR “career progression” OR editor* OR collaboration* OR mentor* OR author* OR funding OR scholarship OR grant AND “dental scientist*” OR “dental researcher*” OR “dental research” OR “dental science” OR “dental faculty”. We adapted the search strategy to the requirements of each database.

### Selection of sources of evidence and data charting

Retrieved references were exported to a reference manager software (EndNote Web®). First, duplicate citations automatically identified by the software were excluded, followed by manual identification and deletion of the remaining duplicated references.

Study selection was carried out independently by two reviewers (L.G.R. and M.C.T.). To ensure reliability, the calibration of reviewers was conducted in a pilot test. Inter-examiner agreement was assessed using the Kappa index^16^. Disagreements were resolved by consensus with the reviewers and the research team. Study selection was carried out in two stages: 1) by reading the titles and abstracts (level 1 analysis) and later 2) by screening the full texts (level 2 analysis). Once references were selected from the level 1 analysis, they were exported to the software Rayyan®. The same method was carried out for the updated search.

A data charting form was created using Microsoft Office Excel® for MAC (version 16.78.3). After approval of the data extraction tool by all authors, two reviewers extracted data independently; in case of disagreements, a third reviewer was consulted. The following variables were retrieved from the included articles: authorship, year of publication, country, study design, sampling, gender assessment, intersectionality lens, outcome, and results.

### Synthesis of results

The main focus of the synthesis was to describe the characteristics of the included studies and their results narratively and using visual resources, such as tables and graphs. We aggregated the data of the included studies, and the gender distribution was analyzed in the following contexts: first author gender, last author gender, and faculty member gender distribution. The software Statistical Package for the Social Sciences (SPSS for MAC, version 25.0; IBM Corp., Armonk, N.Y, USA) was used to carry out the descriptive analysis. For this scoping review, a critical assessment and risk of bias analysis were unnecessary, given that the objective was to map the existing evidence.

## Results

### Study selection

**Figure 1:**
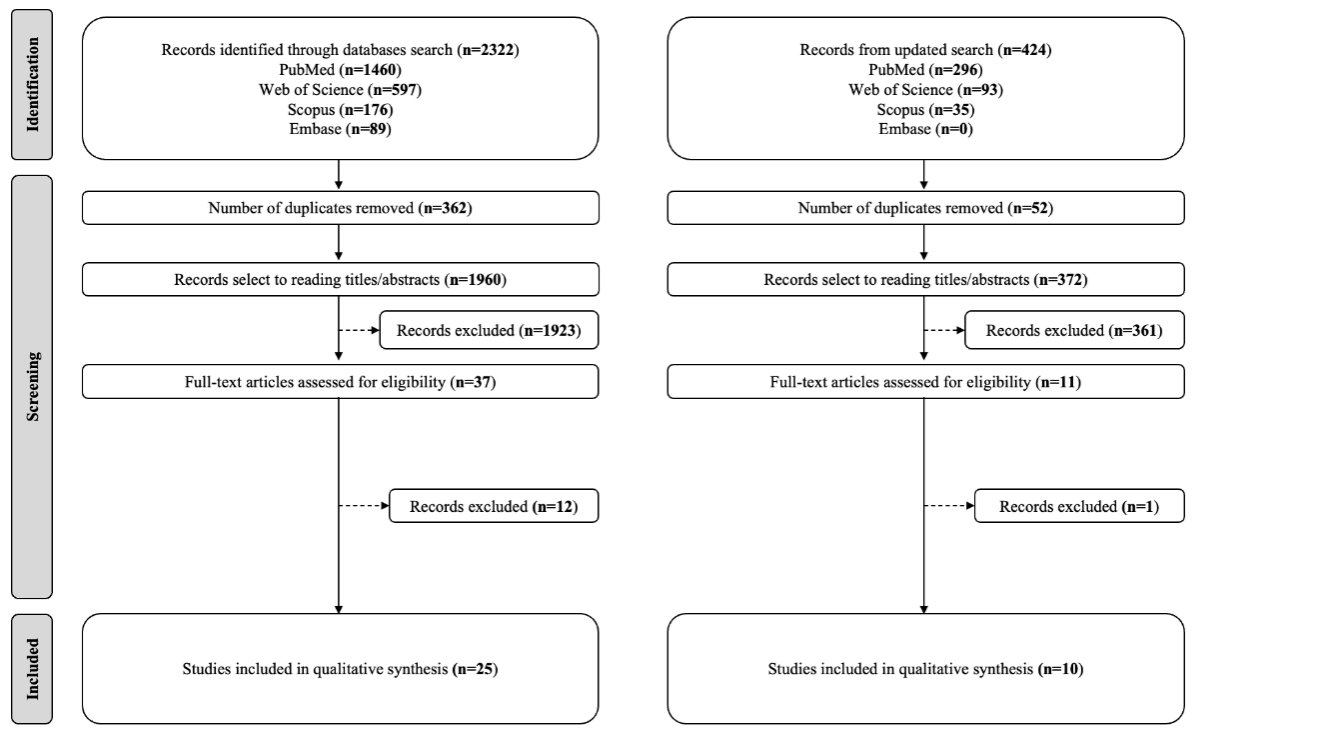
Flowchart with the results of the searches

The initial search carried out in February 2025 returned 2,322 references (Figure 1). After screening, 362 duplicate hits were removed, and 1,923 records were excluded because titles and abstracts contained information that did not meet the eligibility criteria. Interexaminer agreement between reviewers was high (kappa = 0.931). A total of 37 articles were submitted to full-text assessment, and 25 references were included in the qualitative synthesis (Figure 1). For the updated search carried out in June 2025, 424 references were retrieved, with 52 duplicates identified and removed. The titles and abstracts of the remaining 372 references were assessed regarding the inclusion criteria, and 11 were selected for full-text screening, of which 10 were included in the qualitative synthesis. Combining the two searches, a total of 35 references were included in this scoping review. Reasons for exclusions of the 13 references after full-text review are provided in the Supplementary File.

### Study characteristics

**Figure 2:**
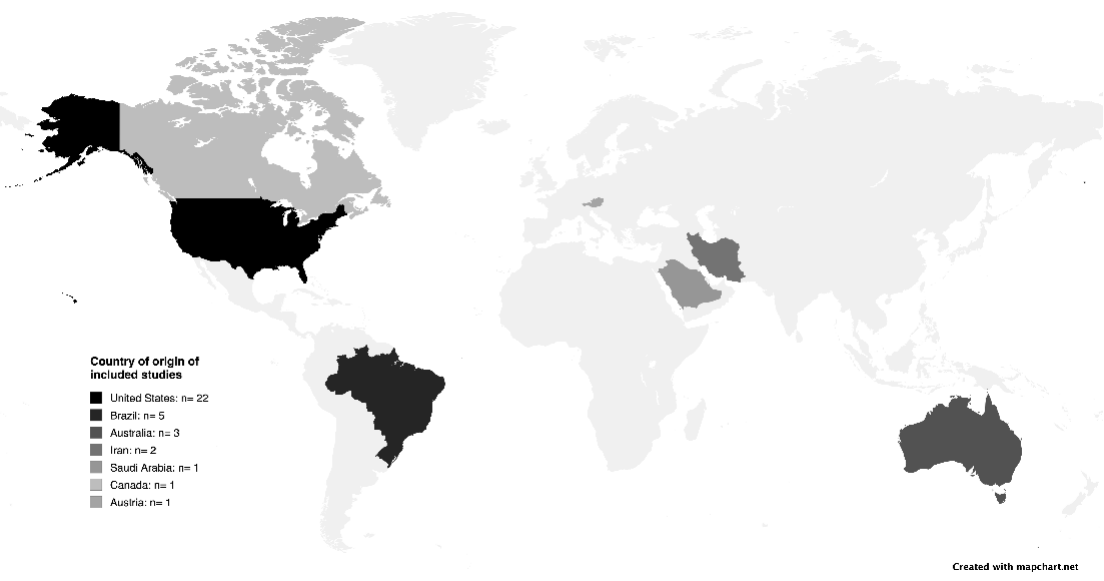
Country of Origin of the included studies

The studies included in this review were conducted by authors from multiple countries: Brazil, the United States, Canada, Saudi Arabia, Iran, Australia, and Austria (Figure 2), and were published between 1998 and 2025 (Table 2). All studies were in English, with 23 published after 2020. The most common study design was cross-sectional (21 studies), followed by bibliometric studies (13 studies), and one longitudinal study. Of the 35 studies, 23 were not open-access publications.

The sample size varied widely, depending on the study's focus, ranging from 100 to 7,104 papers, submissions, journals, editorial boards, and faculty members, depending on the unit of analysis. Gender was assessed in various ways. Three studies did not specify how gender was determined, ten relied on self-reported data from surveys, and the remaining used online searches or assessments based on first names/pronouns. Additionally, nine studies used software to assist in gender identification.

Table 2. Data extraction of the included studies.

**Table 2:** Data extraction of the included studies

Author and publication year, country, and study design	Open Access	Sampling	Gender assessment	Intersectionality lenses	Context	Results	Date of search
Gatti-Reis et al. 2023; Brazil;Bibliometric study	Yes	n= 100 papers; n=407 authors	Assessment of first names with the software GenderAPI® (version 3.14) with a cutoff point of 85% was performed. Manual searches in the website of the academic institution where the author was affiliated, in professional websites (ResearchGate, LinkedIn), social media (Twitter, Facebook), and in the individual author's curriculum vitae were also performed. Corresponding authors of included papers were emailed for assistance. This process was double-checked.	Gender binary variable	Authorship in scientific papers	Proportion by gender: regardless of authorship role, there were 4.8 male authors for each female; among lead authors, 11.3 males for each female; and among senior authors, seven males for each female. All authors: female n=68/407; males 326/407; Lead authors: female n=8/100; male n=91/100; Intermediate authors: female n=50/225; male n=165/225; Senior authors: female n=10/82; male n=70/82	February 2024
Ioannidou & Rosania, 2015; USA; Cross-sectional study	Yes	n= 83 journals; n= 3,060 editorial board members	First name recognition, individual internet search to identify gender, and contact with the journal’s editor-in-chief	Gender binary variable	Participation in editorial and advisory boards of dental journals	All editors: female n=452/3,060 (14.8%); Editor-in-chief: female n=2/83 (2.5%); Associate-editor-in-chief: female 16.0%	February 2024
Bennie & Koka, 2021; USA; Cross-sectional study	No	n= 91 dental and medical journals; n=100 editors	First name recognition, picture identification on the journal website, organization websites, Research Gate profiles, Open Research and Contributor ID (ORCID) profiles or Publons profiles.	Gender binary variable	Participation as associate editors and representation as editors relative to the population from which editors would be chosen - members of the IADR	Editors: female n=15/100 (15.0%); male n=85/100 (85.0%); IADR members: female n=3448/8333(41.38%); male n=4438/8333 (53.26%)	February 2024
Bennie & Koka, 2022; USA; Cross-sectional study	No	n= 28 prosthodontic journals; n=32 editors	Search of the journal's website, the author's first name, and the picture assessment	Gender binary variable	Participation as chief editors of journals publishing prosthodontic science and gender distribution among members of the IADR and the International College of Prosthodontists (ICP)	Editor-in-chief: female n=4/32 (12.5%); male n=28/32 (87.5%); IADR members: female n=3448/8333(41.38%); male n=4438/8333 (53.26%); ICP members: female n=248/906 (27.40%); male n=658/906 (72.60%)	February 2024
Author and publication year, country, and study design	Open Access	Sampling	Gender assessment	Intersectionality lenses	Context	Results	Date of search
D’Silva et al. 2019; USA; Cross-sectional study	No	n= 618 awardees	Not mentioned	Gender binary variable	Recipients of IADR Distinguished Scientist Awards	Awardees: female n=82/618 (13%); male= 536/618 (87%)	February 2024
Phasuk et al. 2021; USA; Cross-sectional study	No	n= 10 organizations; presidents	First name recognition, picture identification, and search of biographical information on the internet	Gender binary variable; Ethnicity/Race	Presidents of selected prosthodontic organizations over the past 20 years	Presidents: female n=20/200 (10%); Number of nonwhite Presidents: 15/200 (7.5%)	
Sartori et al. 2021; Brazil; Bibliometric study	No	n= 3365 first author papers: n= 3398 last author papers	First name recognition. Manual searches in the website of the academic institution where the author was affiliated, in professional websites, social media, and databases (Scopus, PubMed). Assessment of first names with the software Genderize®, with a cutoff point of 85%, was performed.	Gender binary variable	Authorship in scientific papers	First authors: female n=1252/3365 (37.2%); male n=2113/3365 (62.8%); Last authors: female n=768/3398 (22.6%); male n=2630/3398 (77.4%)	February 2024
Alkadi et al. 2021; Saudi Arabia; Bibliometric study	Yes	n= 625 papers	First name recognition. Description was unclear, but the authors stated "Two researchers categorized the papers anddetermined Arabic authors’ gender and affiliations. Gender identification for other authors involved collaboration with Indian and Canadian authors faculty."	Gender binary variable	Authorship in scientific papers	Authors: female n=882/2240 (39%); male n=1358/2240 (61%)	February 2024
Franco et al. 2022; Brazil; Bibliometric study	Yes	n= 4726 submissions	Assessment of first names with the software Genderize®, with a cutoff point of 90%, was performed. Individual searches on the website of academic affiliation, social media, and databases (Scopus, PubMed). For Brazilian authors, the Lattes CV platform was also used (https://lattes.cnpq.br/)	Gender binary variable	Authorship in submitted papers	Before COVID-19 First authors: female n=1355/2222 (61%); male n=867/2222 (39%); Corresponding authors: female= 1237/2210 (56%); male n= 973/2210 (44%); Last authors: female n=1036/2185 (47.4%); male n=1149/2185 (52.6%) During COVID-19 First authors: female n=1389/2400 (57.9%); male n=1011/2400 (42.1%); Corresponding authors: female= 1319/2402 (54.9%); male n= 1083/2402 (45.1%); Last authors: female n=1145/2385 (48%); male n=1240/2385 (52%)	February 2024
Author and publication year, country, and study design	Open Access	Sampling	Gender assessment	Intersectionality lenses	Context	Results	Date of search
Garcia et al. 2020; USA; Cross-sectional	No	Applicants from the National Institute of Dental and Craniofacial Research (NIDCR): n= 3444; NIDCR awardees: n= 1303; National Institutes of Health (NIH) applicants: n= 1,814; NIH awardees: n= 744	Self-reported data collected from the survey	Self-reported by the applicants: male, female, withheld, and unknown (both excluded from the study)	Funding obtained from the National Institute of Dental and Craniofacial Research or the National Institutes of Health (NIH)	NIDCR applicants: female n= 1106/3444 (32%); male= 2338/3444 (68%); NIDCR awardees: female n= 431/ 1303 (33%); male= 872/1303 (67%); NIH applicants: female= 630/1814 (35%); male= 1184/1814 (65%); NIH awardees: female n=255/744 (34%); male= 489/744 (66%)	February 2024
Haag et al. 2023; Australia; Bibliometric study	Yes	n= 165467 all articles; n= 1000 random sample	Picture identification. Authors' full names were searched in Scopus, PubMed, Google Scholar, and ResearchGate, along with the websites of institutional affiliations. An online software (https:// genderize.io/)with a cutoff point of ≥90% of probability was used	Gender binary variable	Authorship in scientific papers	Random 1000 articles first author: female n= 268/944 (28.4%); male= 676/944 (71.6%); Random 1000 articles last author: female n= 207/935 (22.1%); male= 728/935 (77.9%); Top 1000 most-cited articles first author: female n= 197/973 (20.3%); male= 776/973 (79.8%); Top 1000 most-cited articles last author: female n= 157/973 (16.1%); male= 816/973 (83.9%)	February 2024
Kongkiatkamonet et al., 2010; USA; Cross-sectional study	No	n=1202 papers	First name recognition, individual internet search to identify gender, and the affiliated institutional website	Gender binary variable	Authorship in scientific papers and leadership in prosthodontics organizations	First authors: female n=72/451 (16%); Last authors: female n=26/323 (8%); Female presidents of prosthodontics organizations: American Board of Prosthodontics = 0/58; American College of Prosthodontists= 1/38 (3%); American Academy of Fixed Prosthodontics= 1/58 (2%); Academy of Prosthodontics= 1/87 (1%)	
Author and publication year, country, and study design	Open Access	Sampling	Gender assessment	Intersectionality lenses	Context	Results	Date of search
Jones, 1998; USA; Cross-sectional study	No	n= 833 faculty members	Self-reported data collected from the survey	Gender binary variable	Faculty research productivity (total number of articles published in refereed journals and book chapters published during their full-time academic careers)	Faculty members: female= 128/833 (15.4%); male= 705/833 (84.6%); Mean number of publications: female= 6.5 SD=8.5 (P<0.001); male= 11.6 SD=8.5 (P<0.001); External funding: female=$73,597 SD=217,685 (P<0.03); male= $392,765; SD=217,685 (P<0.03)	February 2024
Karhade; Middleton; Simon, 2019; USA; Cross-sectional study	Yes	n= 184 faculty members; n=184 publications	Identification from pronoun/photo on web page of institutional affiliation, or by gender as listed on other websites (i.e., Healthgrades)	Gender binary variable	Promotion and academic productivity within academic pediatric dentistry	Faculty members: female= 90/184 (48.9%); male= 94/184 (51.1%); Mean number of all PubMed publications: female= 7.00 (95%CI 4.3-9.8), p=0.10; male= 12.80 (95%CI 8.2-17.3), p=0.10; First author publications: female= 2.10 (95%CI 1.3-3.0), p=0.06; male= 4.2 (95%CI 2.6-5.8), p=0.06; Last author publications: female= 1.60 (95%CI 0.7-2.4), p=0.03; male= 3.5 (95%CI 1.9-5.1), p=0.03; Years since graduation - Instructor: female= 10.60 (95%CI 5.6-15.5), p=0.04; male= 35.30 (95%CI 14.3-56.4), p=0.04; Assistant professor: female= 14.60 (95%CI 11.6-17.6), p=0.01; male= 25.00 (95%CI 19.1-31.0), p=0.01; Associate professor: female= 24.60 (95%CI 17.7-31.4), p=0.39; male= 29.80 (95%CI 23.7-35.9), p=0.39; Professor: female= 33.00 (95%CI 28.3-37.7), p=0.72; male= 33.40 (95%CI 27.6-39.9), p=0.72	February 2024
Author and publication year, country, and study design	Open Access	Sampling	Gender assessment	Intersectionality lenses	Context	Results	Date of search
Kim et al., 2024; USA; Longitudinal study	No	Not mentioned	ADA and ADEA's websites	Gender binary variable	The number of women who occupy the upper echelons of academic rank and title	Women’s ratios during academic year 2011-2012 through year 2018-2019: First-year dental student enrollment: 45.8-50.9; Dental school graduates: 45.5-N/A; Dental specialty graduates: N/A-29.5; Practicing dentists: 75.0-67.7.; Full-time faculty: 35.6-40.5; Assistant deans: 35.8-50.6; Associate deans: 35.4-36.8; Deans: 21.1-21.8; Directors: 33.9-38.2; Department chairs: 19.9-28.3; Assistant professor: 45.6-46.7; Associate professor: 34.9-37.4; Instructors: 59.4-64.8; Lecturers: 60.9-55.2; Professors: 18.6-25.7	February 2024
Li et al., 2019; Canada; Cross-sectional study	No	n= 12 Canadian provincial/territorial associations; n= 67 US dental schools; n= 10 Canadian dental schools; Oral health journals	Data on men's and women's membership and leadership in the Canadian Dental Association and the American Dental Association. Contacted Canadian associations by email, asking for the number of women dentists. Data on the sex of the Editor in Chief from the websites or head offices.	Gender binary variable	Women’s representation in leadership positions in North American dental and specialty associations/organizations, dental education, and dental journals, as well as the proportion of men/women researcher members of the American Association for Dental Research.	Members in organized dentistry Canada: female= 8,841/22,226 (39.8); Members in organized dentistry US: female= 33,942/117,677 (28.8); Women leaders Canada: female= 3/12 (39.8); Women leaders US: = 5/24 (20.8)	February 2024
Milgrom et al., 2008; USA; Cross-sectional study	No	n= 294 members of the IADR Behavioral Sciences and Health Services Research Group; n=177 respondents	Self-reported data collected from the survey	Gender binary variable	Number of self-reported published articles in PubMed in the preceding twenty-four months	Respondents: Number of published research articles (Gender): Beta: 0.273 (p=0.003). Authors stated that "Gender and time in research were the best predictors of research productivity of this population. There was no difference in time for research between the men and women in this study."	February 2024
Author and publication year, country, and study design	Open Access	Sampling	Gender assessment	Intersectionality lenses	Context	Results	Date of search
Moreno et al., 2023; Brazil; Bibliometric study	No	n= 100 papers; n=187 authors	Assessment of first names with the software Genderize®, with a cutoff point of 80%, was performed. For those who scored below this mark, the gender was checked in the candidate's online CV (e.g., ORCID, university websites, Research Gate).	Gender binary variable	Authorship in scientific papers	First authors: female n= 15/100 (15%); male n=85/100 (85%); Last authors: female n= 11/87 (12.6%); male n=76/87 (87.4%)	February 2024
Nesbitt et al., 2003; USA; Cross-sectional study	No	n= 870 faculty members	Self-reported data collected from the survey	Gender binary variable; Ethnicity/Race; Family background (marital status/parenthood)	Professional experiences and perceptions of the workplace	Faculty members: female= 257/870 (34.8%); male= 481/870 (65.1%); Ethnicity/Race: white= 608/870 (82.3%); non-white= 262/870 (17.7%); Marital status: female=178/257 (69.3%); male=433/481 (90%); Motherhood= 147/257 (57%); Fatherhood= 423/481 (87.9%); Grant suport: female= 51/257 (19.7%); male= 97/482 (20.1%).Publications: female= 16.6; male= 33.5 p=.000.	February 2024
Nkenke et al., 2015; Austria; Bibliometric study	No	n= 1412 papers	First name recognition	Gender binary variable	Authorship in scientific papers	Ratio of male to female first and last author: 1980=42.3:1; 1990=12.1:1; 2000= 10.1:1; 2010: 4.5:1	
Rajendran et al., 2021; USA; Bibliometric study	Yes	n= 69 pre-prints; n= 69 papers	Assessment of first names with the software Genderize®, with a cutoff point of 51%, was performed. For those who were uncertain, authors performed an internet search and/or a search of the affiliated institutional website	Gender binary variable	Authorship in scientific papers and preprints	Pre-print: First authors: female n= 29/69 (42%); male n=40/69 (58%); Last authors: female n= 23/69 (33.3%); male n=46/69 (66.7%); Papers: First authors: female n= 44/69 (63.8%); male n=25/69 (36.2%); Last authors: female n= 43/69 (62.3%); male n=26/69 (37.7%)	February 2024
Author and publication year, country, and study design	Open Access	Sampling	Gender assessment	Intersectionality lenses	Context	Results	Date of search
Simon et al., 2019; USA; Bibliometric study	Yes	n= 702 faculty members	Assessment of the pronoun, photo on the institutional website, or as it appeared on other websites	Gender binary variable	Authorship in scientific papers, citations	Faculty members: female= 256/702 (36.5%); male= 446/702 (63.5%). Mean number of all PubMed publications: female= 20.4 (95%CI16.3-24.6), p=0.02; male= 12.80 (95%CI 8.2-17.3), p=0.10; male= 20.4 (95%CI 16.3-24.6), p=0.02; male= 34.0(95%CI 28.6-39.5), p=0.10; First author: female= 4.6 (95%CI 3.7-5.5), p=0.25; male= 7.0 (95%CI 5.8-8.1), p=0.25; Last author: female= 5.8 (95%CI 4.2-7.4), p=0.0005; male= 12.1 (95%CI 9.3-14.9), p=0.0005; Mean years since graduation - Instructor: female= 14.2 (95%CI 11.0-17.4), p=0.0041; male= 22.1 (95%CI 18.4-25.8), p=0.0041; Assistant Professor: female= 18.7 (95%CI 16.2-21.2), p=0.006; male= 18.7 (95%CI 16.2-21.2), p=0.006; male= 24.8 (95%CI 22.3-27.3), p=0.006; Associate Professor: female= 25.5 (95%CI23.0-28.1), p=0.008; male= 31.8 (95%CI 28.8-34.8) p=0.008; Professor: female= 29.1 (95%CI 29.1-33.4), p=0.003; male= 35.6 (95%CI 33.9-37.4), p=0.003.	
Weinstein et al., 2022; USA; Cross-sectional study	No	n= 61 deans	Self-reported data collected from the survey	Gender binary variable; race/ethnicity	Participation as deans	Gender: female n= 16/60 (26.7%); Ethnicity/race: white= 46/59 (78%); nonwhite=13/59 (22%)	February 2024
Yuan et al., 2010; USA; Bibliometric study	No	n= 7104 papers; n= 5773 authors	First name recognition, individual internet search to identify gender, and the affiliated institutional website	Gender binary variable	Authorship in scientific papers, deanship of USA dental schools	First authors: female n=463/3556 (13%); male n= 3093/3556 (87%); Last authors: female n=200/2217 (9%); male n=2017/2217 (91%) Tenured female faculty members:1995-96:10%; 2007-08:17%; Female deans: 1985-86=1/58 (2%); 1990-91=3/55 (5%); 1995-96=1/54 (2%); 2000-01=4/56 (7%); 2005-06= 10/56 (18%);	February 2024
Author and publication year, country, and study design	Open Access	Sampling	Gender assessment	Intersectionality lenses	Context	Results	Date of search
Assari & Ahmadyar, 2009; Iran; Bibliometric study	No	n= 1994 papers	Not mentioned	Gender binary variable	Authorship in scientific papers	Corresponding author: female= 664/1994 (33.3%); male= 1330/1994 (66.7%)	February 2024
Gottlieb et al., 2024; USA; Bibliometric study	No	n=2935 articles, 2775 reviewers, four editors in chief, 85 editorial board members	Gender identification based on the first name, according to the GenderAPI software, first name recognition, individual internet search to identify gender, and the affiliated institutional website	Gender binary variable	Authors, reviewers, and members of the editorial board	Authors: female= 2789/7558 (36.9%); male= 4769/7558 (63.1%); reviewers: female= 1779/7416 (23.9%); male= 5637/7416 (76.1%); members of the editorial board: female editor-in-chief: 1/4(25%); female editorial board members: 24/89 (34%)	June 2025
Kaste & Yuan, 2025; USA; Cross-sectional study	No	n= 65 deans	Photo and name recognition	Gender binary variable; ethnicity (White, historically underrepresented- represented minority (URM): Black/Hispanic or other culturally diverse group)	Participation as deans	Deans: female= 19/65 (29.2%); male= 46/65 (70.8%); Gender x cultural and linguistical diversity: female: white= 31.1%; URM= 62.3%; male: white= 68.9%; URM= 37.5%. Of the 65 deans included in the study, the authors reported a male-to-female ratio of 2:1.	June 2025
Lalloo, 2024; Australia; Cross-sectional study	No	n= 48 staff members, 15 board of directors members	Gender identification based on photographs and the Genderize software	Gender binary variable	Participation as leaders of oral health organizations	Headquarters staff members: female= 37/48 (77.0%); Leadership: IADR: female= 5/15 (33%); FDI: female= 8/15 (53%); IFDH: female= 5/5 (100%); Past presidents: IADR: female= 11/100 (11%); FDI: female= 6/47 (13%); Leadership of global dental specialist organisations: female= 5/16 (31%); Global chief dental offices: male= 86/159 (54%)	June 2025
Author and publication year, country, and study design	Open Access	Sampling	Gender assessment	Intersectionality lenses	Context	Results	Date of search
Madhu et al., 2025; USA; Cross-sectional study	Yes	n= 66 U.S.dental schools	The study utilized data from the American Dental Education Association assessment of Dental School Fac- ulties from the years 2011-2024	Gender multiple variable	Participation as academic staff	Women in Academia: 36.2%; women in assistant professor positions: 49.9%; women in senior academic rank positions: women are underrepresented compared to men (PRs ranging from 0.60 to 0.80, as reported by authors).	June 2025
Sofi-Mahmudi et al., 2024; Iran; Bibliometric study	Yes	n= 1850 faculty members	Gender identification based on the first name, when in doubt, authors reported having contacted a student from the dental school to check	Gender binary variable	Authorship in scientific papers	Faculty members with publications: female= 1104/1850 (59.7%); Publications: female= 6255/12838 (48.7%), male= 6583/12838 (51.3%); Citations: female= 39559/99969 (39.6%), male= 60410/99969 (60.4%)	June 2025
Lalloo & Borrell, 2025; Australia; Cross-sectional study	Yes	n= 99 awardees, 113 fellowships	Gender identification based on photographs and the first name, according to the Genderize software	Gender binary variable	Participation among awardees	Distinguished scientist award: female= 35/99 (35.4%); fellowships: female= 66/113 (59%)	June 2025
Wilkinson et al., 2025; USA; Cross-sectional study	Yes	n= 32 dental schools	Self-reported data collected from the survey	Gender multiple variable	Participation in academic leadership positions	Senior adminisrative positions: female= 77/160 (48.1%); Dean: female= 11/32 (34.4%); Clinical dean: female= 11/32 (34.4%); Dean of Student Affairs: female= 23/32 (71.9%); Dean of Academic Affairs: female= 19/32 (59.4%); Dean of Research: female= 13/32 (40.6%)	June 2025
Fleming et al., 2024; USA; Cross-sectional study	No	n= 5066 faculty members	Self-reported data collected from the survey. The study utilized data from the American Dental Education Association 2018-2019 assessment of Dental School Fac- ulties	Gender binary variable and ethnicity	Participation in academic leadership positions	Full-time faculty members: female= 77/160 (48.1%); Minoritized women (13.4%), white women (20.6%). Full-time faculty members (by ethnicity/gender): Asian (5.8%), Black (2.7%), Hispanic (4.6%), Native Hawaiian or Other Pacific Islander women (0.1%), American Indian or Alaska Native women (0.1%).	June 2025
Author and publication year, country, and study design	Open Access	Sampling	Gender assessment	Intersectionality lenses	Context	Results	Date of search
Istrate et al., 2024; USA; Cross-sectional study	No	n= U.S. dental schools	Self-reported data collected from the survey. The study utilized data from the American Dental Education Association (ADEA) 2018-2019 assessment of Dental School Fac- ulties and 2021-2022 ADEA Dental School Faculty Salary and Demographic Census	Gender binary variable and ethnicity	Participation in academic leadership positions	Faculty members: female= 4 in 10 faculty members (40%); Deans: female= 47%; Tenured faculty: female= 17%	June 2025
Ferreira et al., 2025; Brazil; Cross-sectional study	No	n= 196 professionals	Not mentioned	Gender binary variable	Participation as researchers	Researchers: female= 117/197 (59.4%); Published articles: female= 7068/14608 (48.4%), male= 7540/14608 (51.6%); Citations: female= 106703/251257 (42.5%), male= 144554/251257 (57.5%)	June 2025

**Figure 3:**
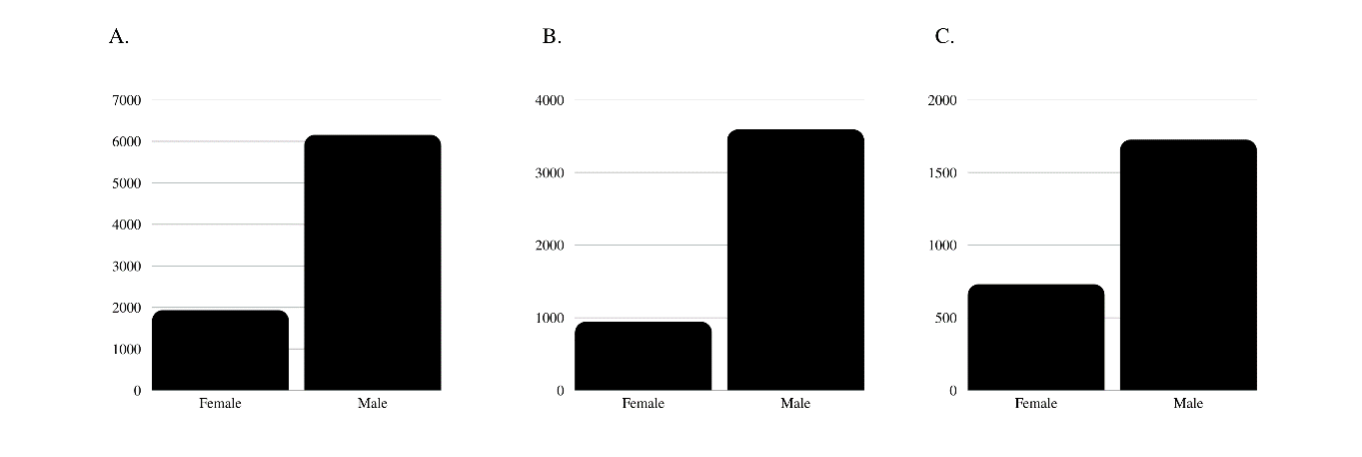
A. First author gender distribution; B. Last author gender distribution; C. Faculty member gender distribution

Six studies explored inequality through an intersectional lens, collecting complementary sociodemographic data, such as race/ethnicity and family status (marital status/parenthood). Gender was treated as a multiple variable in 3 studies. Over time, the context most frequently addressed by the papers was authorship in scientific papers (n=14), faculty members (n=9), and participation as editors (n=3). Figure 3 shows the overall distribution of gender inequality in the variables first author gender, last author gender, and faculty member gender distribution. For first authors, there were about three male authors for each female (3.18). Among the last authors, there were almost 4 (3.97) male authors for each female. As for faculty members, there were around two males for each female (2.36).

Table 3 shows the distribution of the first and last authors by gender from the included articles, according to the independent variables (N=35). Among the 35 first authors identified, 10 (28.6%) were from the South hemisphere, among whom seven (70.0%) were female, and three (30%) were male. The remaining 25 authors were from the North hemisphere; 18 female (72.0%), and seven male (28.0%). Because single-authored papers were considered only in the first author analysis, the last authorship analysis included 33 studies. Among them, six authors were from the South hemisphere, two of whom were women (33.3%), and four men (66.6%). In the North hemisphere, 20 authors were female (74.1%), while seven were male (25.9%).

From the studies included in this review, it was possible to identify the following contexts related to the topic of interest: authorship, academia, participation in the editorial boards of dental journals, funding, recognition, and organized dentistry. 

**Table 3:** Distribution of the first and senior authors by gender from the included studies, according to the independent variables (N=35)

		First author gender **				Last author gender**	
Variable/Category	Total n* (%)	**Female *n* (%)**	**Male *n* (%)**	Total n* (%)	**Female *n* (%)**	**Male *n* (%)**
First author's origin						
South hemisphere	10 (28.6)	7 (70.0)	3 (30.0)	9 (28.1)	3 (33.3)	6 (66.7)
North Hemisphere	25 (71.4)	18 (72.0)	7 (28.0)	23 (71.9)	14 (60.9)	9 (39.1)
Last author's origin						
South hemisphere	6 (18.2)	5 (83.3)	1 (16.6)	6 (18.2)	2 (33.3)	4 (66.7)
North Hemisphere	27 (81.8)	20 (74.1)	7 (25.9)	27 (81.8)	20 (74.1)	7 (25.9)

* Percentage distribution for the columns

** Percentage distribution for the rows.

Because single-authored papers were considered only in the first author analysis, the last authorship analysis included 33 studies.

### Authorship: first, intermediate, last, and corresponding authors

As highlighted by Sartori and colleagues, when people think of a scientist, there is a high probability that the figure that first comes to their mind is of a white male^11^. In this way, by assessing the authorship of scientific papers, it is possible to learn more about female scientists’ contributions. Thus, Sartori and colleagues evaluated the contribution of female authors to publications from dental journals with high impact, and found that male scientists were more frequently authors of papers from high-impact journals in any authorship order^11^.

However, another important variable that may impact female participation is the citation of a given article. Three studies sought to assess female contributions in the most-cited articles in dental research^17^ ^,^ ^18^ ^,^ ^19^. Among the most-cited papers in dental research, female authors remained underrepresented as first and last authors in two studies^17^ ^,^ ^18^. Moreover, Haag and colleagues reported that the representation of women among the most cited articles should be similar to the female representation among authors in other articles from the same database, as the authors' gender should not be taken into consideration when choosing to cite an article^19^. It was concluded that gender inequalities in authorship were observed among first and last authors in general and also in the most-cited publications^19^.

Other specific situations may also impact the representation of women as authors of scientific papers. One study sought to analyze dental research in Iran and found that females were assigned as correspondent authors in about 33.3% of the studies assessed^20^. Six years later, another bibliometric study highlighted inequalities in female representation among authors in oral and maxillofacial literature^21^. Conversely, Rajendran and colleagues highlighted greater representation of female authors in publications that had been peer-reviewed, when compared to female contributions in pre-print publications^22^. Indeed, another study highlighted an increase in the number of women as authors in recent years in a specific journal; interestingly, 48% of the publications assessed had collaborations of authors from both sexes^23^. In the unique scenario of the COVID-19 outbreak, the pandemic may have negatively impacted the submission of articles by female scientists^24^. For first authors, there was a negative impact on the number of submissions from female researchers in comparison to before the COVID-19 pandemic^24^. One study assessed female representation in the Journal of the American Dental Association and found that the representation of female authors has increased over the years, reaching 47% in 2022; however, the authors noted that women remained underrepresented in editorial boards^25^.

### Academia: faculty, productivity, and deanship

The participation of women in Academia has been addressed by several studies, highlighting the need for greater representation among different levels of faculty positions, inequalities in academic productivity, and deanship. Kim and colleagues carried out a study to assess the number of female academics in higher ranks as compared to female students graduating in dentistry^26^. There has been a marked increase in female participation among dental school graduates and academics in North America; however, the majority of female academics are mainly at the instructor level^26^. In addition, a study investigating full-time dental faculty members in the United States highlighted marked race/ethnicity inequalities, as 82.3% of respondents were white.^26^ Women of color represented 13.4% of all full-time faculty members, while white women represented 20.6% of the full-time faculty^28^. Istrate and colleagues noted that comparing the data from 2018-2019 and 2021-2022, faculty members from dental schools were younger, with more participation of female researchers^29^. More recently, Madhu and colleagues noted that women were 36.2% of the faculty members, with a growth from 32.9% in the years 2011-2012 to 43.8% in 2022-2023^30^. 

Yuan and colleagues highlighted that, to advance in academia, scholarly output is a relevant measure^31^. The assessment of the effect of gender on productivity among academics has been a topic of interest in several studies^31^ ^,^ ^32^ ^,^ ^33^. The first authors to mention the relationship of gender on productivity among researchers were Jones and colleagues, who assessed productivity among dental faculty members in schools in Canada and the USA and found that male academics had more publications than female academics^32^. Interestingly, male academics also received more external funding for research than their female colleagues^32^. The trend for a lower number of publications among female academics was also highlighted by Simon and colleagues, who noted that this may partially explain the lower likelihood of female academics being appointed to higher academic positions^33^. There is an underrepresentation of female academics in every rank observed, with the exception of the instructor level, as noted by Kim and colleagues^26^ ^,^ ^33^. However, it is possible that this is not observed across all areas in Dentistry. Karhade and colleagues noted the high representation of women in paediatric dentistry and sought to investigate academic productivity and career progression/promotion in that specific area^34^. Unlike previous studies that assessed dentistry across all specialties, the authors found that productivity was not a factor for faculty rank among pediatric dentistry academics, in addition to no difference between males and females in the number of first or last author publications^34^. Among members of the IADR Behavioral Sciences and Health Services Research Group, the variables gender and time dedicated to research were predictors of productivity^35^. Among the included studies, two assessed academic productivity in specific settings. Sofi-Mahmudi and colleagues assessed publications among Iranian dental faculty and found that while women were the majority of dental faculty members in the country, men exhibited a higher H-index^36^. Another study assessed the profile of Brazilian researchers in Oral Pathology and Oral Medicine and found that 59.4% of them were female. Regarding academic production, no significant differences between male and female researchers were observed^37^.

One measure of leadership in academia is being appointed as faculty dean^28^. Female deans in the United States grew from 2% in the years 1985-1986 to 18% in 2005-06, but female underrepresentation still exists^31^. The proportion of females working as deans has remained low throughout the years, as evidenced by Weinstein and colleagues; in 2021, only 26.7% of dean positions were held by women^35^. The majority of deans in the sample reported race/ethnicity as white (78%), although gender distribution according to race/ethnicity was not reported^38^. Another study assessed the characteristics of dental school deans using an intersectional lens, including historically underrepresented minority groups^39^. The authors reported that most deans were white, while 30.8% of them belonged to the nonwhite group^39^. Of the 65 deans included in the study, the authors reported a male-to-female ratio of 2:1^39^. It must be highlighted that in leadership positions, one study found that the presence of a female dean (n=11, 34.4%) indicated that another administrative position would be occupied by a female^40^.

### Research: participation in the editorial boards of dental journals

The assessment of women’s participation as part of the editorial board of dental journals has been a topic of growing interest. Ioannidou and colleagues highlighted the underrepresentation of women as editorial board members, with only 14.8% of the 69 dental journals included in the analysis having a woman as an editor^41^. Authors also reported differences among different dental specialties, with higher participation of women in dental public health journals in comparison with periodontal and oral and maxillofacial surgery journals^41^. Interestingly, there was more participation of women in the editorial and advisory boards in journals in comparison to women occupying leadership positions^41^. More recently, it was found that the percentage of female researchers as chief editors (15%) was lower than expected in comparison with the gender distribution among IADR members^8^ ^).^ Similar gender inequalities have also been reported among chief editors of prosthodontics journals (12.5%), also lower than the participation of women as IADR members (43.7%)^42^.

### Funding

In this review, the first author to mention funding for scientific research was Jones and colleagues, who noted that male academics were more productive than female colleagues and also received more external funding for research^32^. They also highlighted the need for additional measures by institutions aiming to mitigate the observed inequalities, such as preparing faculty members to apply for grants successfully and fostering partnerships of a research team in different moments of researchers’ careers, from beginner to mid-career and senior scientists^32^. More recently, one study sought to assess the gender distribution of award funding provided by the National Institute of Dental and Craniofacial Research of the National Institute of Health^43^. The authors found that male researchers were the majority among applicants and awardees. However, there was no difference in the award rate according to the gender of the applicant^43^.

### Recognition

In addition to funding, gender inequality has also been reported in recipients of awards^44^. Inequalities in recognition for academic success may adversely impact the careers of researchers and the overall quality of what is produced^44^. However, it has been highlighted that the gender distribution of the IADR distinguished scientist award over time is unequal, as only 13% of women have been recipients of the award^44^. Moreover, between 2019 and 2024, the IADR had 99 awardees for the Distinguished Scientist awards, with 35% of them being female scientists^45^. All of them were from high-income countries: the United States (38%), the United Kingdom (12%), and Australia (11%)^45^.

### Organized dentistry: participation of women as members of organizations

One measure of participation of women in organizations is their presence as members of the IADR^8^. Of the reported 8,333 members of this specific Association, 3,448 are women (41.38%), 4438 are men (53.26%), and 447 did not provide information on gender^8^. Another study found that among the staff of the IADR in its headquarters, females were the majority^46^. However, it must be noted that most of them were from high-income countries in North America and Europe, highlighting country inequalities in female leadership^46^. Moreover, in the assessment of congress locations, 90% took place in countries with a high income^46^. Li and colleagues sought to assess the participation of women in membership and leadership in the Canadian Dental Association and the American Dental Association. In Canada, the leader/member ratio was 0.91; in the United States, the leader/member ratio among women is 0.67^47^. Phasuk and colleagues highlighted the relevance of role models and assessed the number of female and nonwhite presidents of prosthodontics organizations and found that female representation between the years of 2000 to 2019 was only 10%^48^.

The studies in this review also made suggestions on how to address persistent gender inequalities, which were grouped in the following categories: recommendations to dental schools, recommendations to journals, and institutions/funders.

### Recommendations to dental schools

Nesbitt and colleagues highlighted that the need to recognize that the dental school climate is not gender neutral is a good starting point to meaningful change^27^. This was also mentioned by Ioannidou and Rosania^41^. One study suggested that female dental faculty should be supported in applying for grants, in addition to networking and identifying partnerships for research^32^; while others highlighted the relevance of mentorship to female scientists^41^. Leaders should assess the situation at first and then tailor target-specific interventions towards the observed problems^27^. The authors suggested that special care be given to making the academic environment a welcoming one, to guarantee permanency in academia, in addition to providing the student with mentorship and role models for their careers^27^. Another interesting suggestion was to promote networking collaborations with international institutions^20^. Moreover, one study mentioned the relevance of making adjustments in the evaluation of the curriculums of academic mothers, taking into consideration the impact of the early years of motherhood on their productivity^18^. The extra financial burden to academic mothers due to childcare expenses should also be addressed and made more flexible - the authors gave the example of flexible use of grants, such as spending with family or childcare needs^11^. Some authors highlighted the need for structural changes to address the challenges faced by female faculty, with specific goals tailored to address the gender gaps observed^30^. One study mentioned the following strategies to make academic dentistry more diverse: inclusive hiring, with attention to diversity in evaluating committees and candidates providing diversity statements; cluster hiring, where more than one faculty member is hired at once to work together for a specific purpose, providing empowerment and guidance; and Employee Resources Groups, representing communities where employees can be heard and mentored to support their career progression^28^. Kaste and Yuan also recommended the use of standardized metrics to assess diversity, leadership, and female representation in academic settings^39^.

### Recommendations to dental journals

One study recommended that authorship be blinded when submitting a paper to a journal, in an attempt to lessen the observed gender gap in authorship of scientific papers^21^. Furthermore, dental journals should recognize that this is a problem and work towards improving this scenario^21^. As for editors, one study highlighted the relevance of transparency and the need for collaboration between men and women towards improving the gender gap in dental research^8^. It has been noted that it is necessary to increase female representation among authors, but also among editors and reviewers, to promote gender equity in dental research^25^.

### Recommendations to institutions and funders

Sartori and colleagues suggested that peer review should be gender-conscious in the selection processes of funding, in addition to creating and widening the use of platforms that promote identification of female scientists for different purposes, from speakers at conferences to networking opportunities^11^. In addition, one study recommended that the selection committees of funding agencies should have adequate gender representation among their members, with a previous training on unconscious bias, using a transparent process^44^. One study highlighted the intersectionality lens as urgent to assess and recognize the performance of researchers, so as to promote efforts to recognize scientists for their contributions^45^ equally.

## Discussion

In 1948, gender equity was recognized by the United Nations as a human right^49^. This was after the two World Wars, when there was an increased need for females to occupy the labor market^50^. Since then, this topic has been of growing interest to the public, especially in recent years. Although progress has been made throughout the decades, issues related to gender equity remain a matter of concern^51^. The World Health Organization (WHO) in its Agenda of 2030 for Sustainable Development Goals highlights "gender equity" as goal number 5 to be reached by 2030 across the globe^52^.

In research, in the second half of the 20th century, there was also a need for more workers in science^50^. Accordingly, the scenario for gender representation in dental science has changed over the past decades, with growing female representation across the world^6^. However, some inequalities in different domains persist; thus, one worthwhile task is to address the phenomenon to understand better how it manifests^4^. In this way, a scoping review is an ideal method to map the existing evidence and guide future research questions, whose answers will allow for a better understanding of a given topic^13^. In addition, they are a particularly interesting tool to map emerging topics of interest, such as how gender inequality manifests itself in dental research^13^.

In this review, most included studies were written by authors affiliated with institutions in the USA, followed by Brazil, and other countries, such as Saudi Arabia, Australia, Canada, Austria, and Iran. This is interesting as it may influence the population under study. Indeed, in this review, seven studies were carried out with the American population^26^ ^,^ ^27^ ^,^ ^31^ ^,^ ^33^ ^,^ ^34^ ^,^ ^38^ ^,^ ^43^, two studies assessed both the American and Canadian population^32^ ^,^ ^47^, one study assessed American and European organizations^48^, and one study looked into the gender gap in publications of international dental journals based in Brazil^24^. The remaining studies assessed international journals and organizations with participation of researchers from any country. In this way, the findings from this review may reflect the populations that were under study, and as such, more variety in this target population may produce different outcomes, according to different cultures across the world. A difference in gender inequality among authors of top-cited papers across continents was highlighted in one study^17^, while another reported that Latin America exhibited the highest contribution of female authors in the position of first or last authorship^11^. In this review, among the first authors of the included papers, women are the majority regardless of their country of affiliation. However, in the last authorship analysis, men were the majority of the last authors, except for studies from the Northern Hemisphere. The representation of women among last authors has been shown as relevant, as one study saw a higher representation of females as first authors when the last author was a woman^11^. 

A significant part of the studies included in our review were published after 2020, which highlights the recent interest in this topic in dental research. The majority of studies that had addressed gender inequality in dental research were cross-sectional, which allows the assessment of the prevalence of the phenomenon at a point in time with limited inference of causal relationship at its preliminary stages^53^. In addition, the majority of the included papers were not open-access publications. Open access publications present a clear advantage of widely distributing research findings to other researchers and the public at large^54^. However, having research findings behind paywalls limits the access of other researchers and the public, as there is a need for the payment of subscriptions to journals to access the paper^54^, which in turn may limit the widespread dissemination of the paper by its authors. In addition, the publication costs may pose a burden to authors, especially to those of lower socioeconomic status or with limited funding for research dissemination^55^. 

From this review, it has become clear that while women may be the majority among dental school graduates in several countries^5^, they are still underrepresented in dental research. **U**pon analysis of the results obtained, it is noticeable that many contexts of interest were related to career advancement, such as authorship, academia (regarding researchers' productivity), funding, and recognition. This may be explained by the criteria used in academia in incentive and reward systems, including how researchers and their work are assessed^56^ ^,^ ^57^. A global study of promotion criteria in biomedical science faculties found that research metrics-such as publication count and authorship order-still dominate, while other indicators, like citation impact, protocol transparency, and adherence to reporting guidelines, are often overlooked^58^. As for research funding, the two studies that addressed it highlighted that male academics receive more money for research^32^ ^,^ ^44^. However, if male scientists receive more money than their female colleagues, it is possible that this may further widen the gender gap in research, as with the funding they receive, scientists carry out more research, hence increasing their productivity^59^. This echoes the findings from the aggregated data in this review, with persisting gender inequalities among authors, especially among last authors, with almost four male authors for every female.

In science, diversity has been recognized as a key driver of innovation, exceptional performance, and creative thinking, with a significant potential to inspire groundbreaking research and forward-looking ideas^60^. The advocacy for diversity is a matter of social justice and human rights^60^. Gender discrimination and inequalities are not only unfair but also unacceptable^61^.

The findings from this scoping review highlight pervasive gender inequalities in dental research, especially among leadership positions in different spheres. It is important to acknowledge the profound impact that restrictive gender norms and inequality have on women's lives, including the psychosocial challenges they face (61, 62). The WHO has highlighted how these inequalities can negatively affect women's health outcomes^62^. Examples include limited representation in leadership positions, such as those reported in this scoping review; the disproportionate burden of caregiving responsibilities and exposure to subtle, daily discriminatory behaviors in the workplace, which often lead women to question their sense of belonging and professional achievements^62^. One study found that perceived gender discrimination significantly affects women's mental health^61^. This finding underscores the need to tackle discriminatory gender behaviors not only to promote diversity in science but also to protect the mental health and wellbeing of women^61^. In this review, there was heterogeneity in the method of gender identification, with the majority of studies using searches on the internet or assessments of researchers' first names/pronouns. WHO recognizes gender as multidimensional and a non-binary construct^63^; however, most of the research in this topic assessed gender as a binary variable. In this review, this was the case for all but one study, the publication of Garcia and colleagues, in which data for gender were self-reported and collected according to the options of male, female, withheld, or unknown^43^. However, the assessment of gender as a complex and non-binary variable is relevant to fully capture the nuances and complex nature of this construct.

Intersectionality was first introduced by Crenshaw, highlighting the need to consider the cumulative effect of intersecting social identities in the individual experiences of discrimination and oppression^64^. In this way, she described the discrimination of women of color as a result of the intersection between sexism and racism, whose experiences were often marginalized and overlooked^64^. Traditionally, feminism recognized men and women, with no regard to other social identities, which failed to capture the lived experiences of groups with intersecting identities^65^. In this way, Crenshaw proposed the inclusion of social identities beyond gender, but also race, socioeconomic status, and sexuality^64^.

Thus, this review aimed to map the scope and nature of evidence on gender equity and discrimination in Dental research, using intersectional lenses, and we found three studies explored gender inequality according to the intersectionality framework. Nesbitt and colleagues assessed workplace perceptions and lived experiences of dental faculty members from the United States according to gender, ethnicity/race, and family background^27^. While the authors did not find gender inequalities according to race/ethnicity, there was a difference for family background, as women were found to be less frequently married or with a partner than their male colleagues, which was statistically significant (males= 90.0%; females= 69.3%)^27^. In academia, Weinstein and colleagues surveyed dental school deans in the United States, and they collected data on gender and ethnicity; however, the authors reported the prevalence of female and male deans and the ethnicity distribution, but not the gender distribution according to ethnicity, which may limit the interpretation of the results regarding intersectionality^38^. In dental organizations in prosthodontics, one study reported low representation of women and people of color, highlighting the need for increased diversity among the population under study^48^. Indeed, inequalities are often greater among women with intersecting identities, and it is only by assessing their compounding effects on discrimination that it is possible to fully comprehend the representation and challenges of all women in research^3^. In dental research organizations, it has been shown that while female representation is increasing, they are mostly from high-income countries, which limits the representation of the global population among dental organizations and in the pool of dental researchers^46^. In this way, the origin and location of global leaders, in addition to the location of congresses, should take into account the representation of high- and low-income countries for an equitable inclusion of researchers^46^.

Therefore, there is a limitation in the majority of studies addressing gender inequality in dental research - the limited use of intersectionality lenses when assessing female representation. It is time for the scientific community to address sexism and racism in dental science together, in addition to assessing gender as a complex and non-binary category. Future studies should adopt an intersectional lens with self-reported data obtained directly from the researchers. In addition, it would be relevant to assess different areas of dentistry, so as to compare the data obtained from general and specific specialties and design specific interventions when needed. Furthermore, it would be relevant to look at the first step towards an academic career, the dental students, who will choose to be or not to be academics and future leaders in dental research.

There are a number of strengths to this scoping review. First, the search strategy; there was no limitation to language and year of publication in the search, which allows for a more thorough search of the available literature on the topic. In addition, we sought to assess gender inequality in dental research with an intersectional approach, aiming to capture the real challenges faced by female dental researchers across different contexts. Moreover, from the included studies, it was possible to highlight key strategies and recommendations to address persistent gender inequalities across different domains, such as dental schools, academic journals, and institutions/funders.

## Conclusion

This scoping review has demonstrated that gender inequalities in dental research across different levels still persist, particularly in leadership positions. Further evaluations on this topic are encouraged, especially research under an intersectionality lens, on a broader scope of concepts, and testing interventions to effectively approach the observed gender gaps across different levels.

## Data Availability

The research data are available upon request.
